# Does prematurity “per se” cause visual deficits in preterm infants without retinopathy of prematurity?

**DOI:** 10.1038/s41433-023-02384-4

**Published:** 2023-01-25

**Authors:** Olaf Dammann

**Affiliations:** Departments of Public Health & Community Medicine, Pediatrics, and Ophthalmology, Tufts University School of Medicine, Boston, MA USA

**Keywords:** Pathogenesis, Risk factors

Compared to infants born at term, preterm infants are at increased risk for adverse visual outcomes (AVO) such as visual acuity deficits, visual field restrictions, refractive errors, strabism, among others [1]. At least some AVO among children born preterm can be attributed to retinopathy of prematurity (ROP) [2]. However, some preterm infants without ROP also develop AVO. In one study of infants born after the turn of the millennium, overall vision impairments, myopia and astigmatism in particular, were more frequent among preterm infants without ROP compared to term controls [3]. In another study, strabism was present in 12% of children up to 3 years of age who were born preterm, were screened between 2011 and 2018, and had no ROP [4]; the overall prevalence in a community setting is about 6% [5]. Another example is a study of adult individuals between 18 and 52 years born preterm (≤32 weeks GA) that reports a prevalence of amblyopia at examination of 10% among those without ROP, 24% in those with ROP, and only 2% in a term control group [6].

Authors sometimes argue that the presence of long-term AVO among children born preterm who did not have ROP suggests that preterm birth “per se” has an impact on long-term visual outcomes. For example, young adults born with a birthweight <1500 g appear to have lower visual acuity (both near and distance), mean deviation, and contrast sensitivity compared to young adults born at term [7]. Some of these differences remained after exclusion of preterm born individuals who had ROP. The authors speculate that “[t]he reason may be prematurity per se since individuals without previous ROP or neurological complications are also affected” [7] (emphasis mine).

In another study, investigators found that “[e]xtreme prematurity without impact of ROP is associated with increased ganglion cell + inner plexiform layer, outer nuclear layer, and retinal thickness at the foveal centre as well as reduced foveal depth compared to full-term controls at age 6.5. This indicates that prematurity per se may have a profound effect on foveal anatomical maturation during the first months after birth” [8](emphasis mine).

Other examples of studies whose authors attribute the occurrence of AVO to prematurity or preterm birth “per se” include the following (all emphasis mine):

“Function of photoreceptors was affected in prematurely born children, possibly also in children without previous ROP. Whether immaturity per se affects the retinal function remains to be elucidated.” [9]

“Reduced rim area of the optic nerve head was found in preterm children of school age. Previous ROP or neurological complication did not influence the result, suggesting the preterm birth per se was the reason for the reduction. [10]”

The argument that AVO among preterm infants without ROP must be due to prematurity “per se” is as flawed as the argument that fatal car accidents among sober drivers must be due to driving “per se”. I do not think that those who refer to prematurity “per se” do in fact refer to preterm birth as an event that may be considered causally responsible for the occurrence of AVO. Just as driving “per se” does not cause fatal car accidents, preterm birth “per se” does not cause AVO. But what role does preterm birth play in the aetiology of AVO, if not a causal one?

**Fig. 1:**
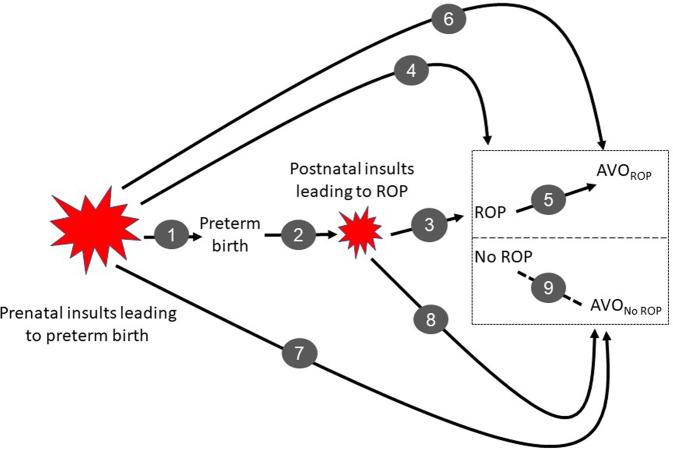
Pre- and postnatal factors that contribute to the occurrence of retinopathy of prematurity (ROP) and adverse visual outcomes (AVO). Certain prenatal scenarios (e.g., preeclampsia, intrauterine infection) are associated with an increased risk for preterm birth (1), which is in turn associated with postnatal insults (2) that contribute to the occurrence of ROP (3). Some prenatal factors also appear to be associated with an increased risk for ROP (4), which in turn increases the risk for some AVO (5). There may also be prenatal factors that contribute to AVO among infants with ROP that are not mediated by ROP (6). If preterm birth “per se” is not a cause but a precondition for ROP and AVO, what are the prenatal (7) and postnatal (8) inducers of AOV in preterm infants without ROP (9)?

Consider the aetiology of ROP and subsequent AVO (Fig. 1). Preterm birth and low birthweight are “the strongest known risk factors for development of ROP” [11]. Retinal vascularization at birth is incomplete in preterm infants. Shortly after birth, the two phases of ROP become apparent with initial reduction/arrest of vessel formation and later with an overshoot and disorganization of vessel growth [12–14]. The main current etiological paradigm invokes postnatal “exogenous stresses […] such as fluctuations in oxygen, oxidative stress, nutritional factors […] activate inflammatory, oxidative, and hypoxic signalling pathways” [12] (Fig. 1, link 3), which in turn affect retinal vasculogenesis, mainly via vascular endothelial growth factor (VEGF) [12]. Another paradigm is that prenatal infection-associated inflammation (Fig. 1, link #4) contributes to an increased ROP risk [15].

It seems reasonable to say that it is not preterm birth “per se”, but its prenatal determinants (Fig. 1, link #1) and postnatal consequences (Fig. 1, link #2) that play a causal role as “inducers” of ROP and, as I suggest here, perhaps also as inducers of AVO in preterm infants with (Fig. 1, link #6) and without ROP (Fig. 1, links #7 and 8). In this etiological scenario, inflammation and growth factors like vascular endothelial growth factor (VEGF) play a role as “mediators” between inducers (oxygen, infection) and ROP/AVO. Immaturity of the infant and their retinal vasculature at birth play the role of a “background condition” (precondition, prerequisite) that paves the way for disease inducers and their mediators resulting in ROP/AVO. In term infants, such insults and mechanisms can also occur and might contribute to an ROP-like retinopathy [16], albeit much less frequently than in preterm newborns. Preterm birth “contributes” to ROP/AVO occurrence as a precondition, i.e., not as inducer or mediator but as a background condition. In other words, the etiological framework summarized in the figure supports my main argument that prematurity “per se” is not causally involved in ROP/AVO aetiology, but only as a non-necessary background condition which is associated with a whole host of potentially causal risk factors for AVO. Thus, while ROP might contribute to AVO (Fig. 1, link #5), AVO among preterm infants without ROP (Fig. 1, link #9) are not due to prematurity “per se” but likely due to a sequence of prematurity-associated prenatal and postnatal factors that have causal (inducing) or mechanistic (mediating) functions (Fig. 1, links #7 and 8), not just that of a background condition. I have outlined the general structure of such etiological (causal-mechanical) explanations in detail elsewhere [17, 18].

In sum, I suggest that preterm birth “per se” should not be considered a cause of AVO in preterm infants without ROP. The main reason is that blaming prematurity “per se” might curtail further research into the causes and mechanisms that explain the occurrence of visual abnormalities in preterm infants without ROP. It might even lead to the conclusion that it is futile to look beyond ROP in preterm infants because with the baby being born preterm the horse is already out of the barn. Infection and inflammation are one set of candidate contributors to AVO occurrence in both children with and without ROP [19]. There is plenty of room for research designed to further test this and other etiological hypotheses if we refrain from attributing to prematurity “per se” what should be attributed to causes and mechanisms associated with preterm birth.
